# Molecular epidemiology of the SARS-CoV-2 variant Omicron BA.2 sub-lineage in Denmark, 29 November 2021 to 2 January 2022

**DOI:** 10.2807/1560-7917.ES.2022.27.10.2200181

**Published:** 2022-03-10

**Authors:** Jannik Fonager, Marc Bennedbæk, Peter Bager, Jan Wohlfahrt, Kirsten Maren Ellegaard, Anna Cäcilia Ingham, Sofie Marie Edslev, Marc Stegger, Raphael Niklaus Sieber, Ria Lassauniere, Anders Fomsgaard, Troels Lillebaek, Christina Wiid Svarrer, Frederik Trier Møller, Camilla Holten Møller, Rebecca Legarth, Thomas Vognbjerg Sydenham, Kat Steinke, Sarah Juel Paulsen, José Alfredo Samaniego Castruita, Uffe Vest Schneider, Christian Højte Schouw, Xiaohui Chen Nielsen, Maria Overvad, Rikke Thoft Nielsen, Rasmus L Marvig, Martin Schou Pedersen, Lene Nielsen, Line Lynge Nilsson, Jonas Bybjerg-Grauholm, Irene Harder Tarpgaard, Tine Snejbjerg Ebsen, Janni Uyen Hoa Lam, Vithiagaran Gunalan, Morten Rasmussen

**Affiliations:** 1 Virus Research and Development Laboratory, Virus and Microbiological Special Diagnostics, Statens Serum Institut, Copenhagen, Denmark; 2 Infectious Disease Preparedness, Statens Serum Institut, Copenhagen, Denmark; 3 Department of Epidemiology Research, Statens Serum Institut, Copenhagen, Denmark; 4 Department of Bacteria, Parasites, and Fungi, Statens Serum Institut, Copenhagen, Denmark; 5 International Reference Laboratory of Mycobacteriology, Statens Serum Institut, Copenhagen, Denmark; 6 Global Health Section, University of Copenhagen, Copenhagen, Denmark; 7 Infectious Disease Epidemiology and Prevention, Statens Serum Institut, Copenhagen, Denmark; 8 Department of Clinical Microbiology, Odense University Hospital, Denmark; 9 Department of Clinical Microbiology, Copenhagen University Hospital Amager-Hvidovre, Hvidovre, Denmark; 10 Department of Clinical Microbiology, Zealand University Hospital, Køge, Denmark; 11 Center for Genomic Medicine, Copenhagen University Hospital Rigshospitalet, Copenhagen, Denmark; 12 Department of Clinical Microbiology, Copenhagen University Hospital Rigshospitalet, Copenhagen, Denmark; 13 Department of Clinical Microbiology, Copenhagen University Hospital-Herlev and Gentofte, Herlev, Denmark; 14 Department for Congenital Disorders, Statens Serum Institut, Copenhagen, Denmark; 15 Clinical Microbiology Department, Aarhus University Hospital, Aarhus, Denmark

**Keywords:** SARS-CoV-2, COVID-19, variant of concern, Omicron, BA.2, BA.1

## Abstract

Following emergence of the SARS-CoV-2 variant Omicron in November 2021, the dominant BA.1 sub-lineage was replaced by the BA.2 sub-lineage in Denmark. We analysed the first 2,623 BA.2 cases from 29 November 2021 to 2 January 2022. No epidemiological or clinical differences were found between individuals infected with BA.1 versus BA.2. Phylogenetic analyses showed a geographic east-to-west transmission of BA.2 from the Capital Region with clusters expanding after the Christmas holidays. Mutational analysis shows distinct differences between BA.1 and BA.2.

Following the discovery of the severe acute respiratory syndrome coronavirus 2 (SARS-CoV-2) variant of concern Omicron (Phylogenetic Assignment of Named Global Outbreak (Pango) lineage designation B.1.1.529) on 19 November 2021 (week 46) in South Africa [1], this variant with immune evasive properties has spread rapidly worldwide [2-4]. Since the Omicron emergence, sub-lineages within Omicron have been described, notably BA.1, BA.1.1 and BA.2 [5,6]. The first Omicron sub-lineage BA.1 expanded rapidly and replaced the Delta variant (Pango lineage designation B.1.617.2) [7]. However, an increasing number of SARS-CoV-2 cases with the Omicron sub-lineage BA.2 have been reported in several countries, especially in Denmark [8]. Here, we provide a molecular epidemiological characterisation of the first BA.2 cases identified in Denmark.

## Omicron sub-lineage BA.2 growth in Denmark

The Danish national SARS-CoV-2 genomic surveillance system [9] identified the first two cases of Omicron sub-lineage BA.1 in samples from 22 November 2021 (week 47; not shown). On 5 December 2021, less than 2 weeks later, the first BA.2 case (week 48) was detected. At this timepoint, BA.1 accounted for only 2.7% of all variants among sequenced samples (Supplementary data S1: Lineage Prevalence). Subsequently, Omicron (BA.1, BA.1.1 and BA.2) rapidly displaced the Delta variant and, by week 5 in 2022, accounted for ca 100% of all sequenced variants in Denmark. The prevalence of BA.1 increased from 2.8% in week 48 to 71.9% in week 51 in 2021, thereafter declining to 7% by week 5 in 2022. The prevalence of BA.2 increased from less than 0.1% to 89.2% of sequenced samples during this 10-week period.

## Characterisation of Omicron BA.2 cases

In total, 16,137 BA.1 and 2,623 BA.2 cases were identified among 55,273 SARS-CoV-2-positive cases confirmed by RT-PCR tests performed at both community testing centres and hospitals between 29 November 2021 and 2 January 2022 (weeks 48–52) with usable consensus genomes (≤ 3,000 Ns) obtained through whole genome sequencing (WGS), as previously described [10]. During the period from 29 November to 16 December 2021, all samples that indicated an Omicron variant based on variant-specific PCR (S:WT452) were selected for WGS. From 17 December 2021 to 2 January 2022, samples from community testing centres were randomly selected for WGS by an algorithm from all positive samples with cycle threshold (Ct) values below 35. Samples from Omicron screening after 20 December 2021 were limited to specific patient groups at some hospitals across the country and were selected based on variant-specific PCR indicating Omicron variants during the study period. The proportion of samples from hospitals were similar for both BA.1 and BA.2 (16% and 18%, respectively, in 4/5 regions). Whole genome sequences have been submitted to the Global Initiative on Sharing All Influenza Data (GISAID) sequence database [11].

Risk ratios (RRs) of hospitalisation with BA.2 vs BA.1 were estimated using a log-linear Poisson regression model, adjusted for sex, age, vaccination status, time period, geographic region, comorbidities and SARS-CoV-2 reinfection, as described previously [12]. No significant differences were observed between individuals infected with BA.1 and BA.2 for age, sex, reinfection or 30-day mortality (Table 1) or for the adjusted hospitalisation RR overall (p = 0.19) or within strata of vaccination status (p = 0.59) (Table 2). When limiting hospitalised cases to those with registered coronavirus disease (COVID-19) diagnoses, the RRs for hospitalisation with BA.2 vs BA.1 remained non-significant (n = 277 hospitalised cases; RR: 1.06 (95% confidence interval (CI): 0.77–1.47)).

**Table 1:** Descriptive statistics of cases with SARS-CoV-2 Omicron variant sub-lineages BA.1 and BA.2, Denmark, 29 November 2021–2 January 2022 (n = 18,760)

Characteristics	Omicron BA.1		Omicron BA.2				p value
	n	%	n		%		
Total cases	16,137	86.0	2,623		14.0		NA
Age							
Median (IQR)	31	31–48	32	21–49		0.0814^a^	
Sex							
Males	7,887	48.9	1,237		47.2		0.10^b^
Females	8,25	51.1	1,386		52.8		
SARS-CoV-2 reinfection							
No	15,167	94.0	2,465		94.0		0.98^b^
Yes	970	6.0	158		6.0		
Deaths related to SARS-CoV-2 infection^c^							
No	16,101	99.8	2,615		99.7		0.42^b^

IQR: interquartile range; NA: not applicable; SARS-CoV-2: severe acute respiratory syndrome coronavirus 2; US: United States.

^a^ Wilcoxon signed-rank test performed in SAS software version 9.4 (SAS Institute, Cary, US).

^b^ Chi-squared test performed in SAS software version 9.4 (SAS Institute, Cary, US).

^c^ A SARS-CoV-2-related death was defined as death within 30 days after a positive SARS-CoV-2 RT-PCR test. Occurrence of death was observed for more than 30 days in all cases by using complete data on death extracted on 22 February 2022 from the Danish national COVID-19 surveillance database.

**Table 2:** Risk ratio of hospitalisation within 14 days after infection with SARS-CoV-2 Omicron variant sub-lineage BA.2 compared with BA.1, overall and according to vaccination status, Denmark, 29 November 2021–2 January 2022 (n = 18,681^a^)

Characteristics	COVID-19 hospitalisation							
	Yes (n = 423)		No (n = 18,258)^a^		RR^b^			
	n	%	n	%	Crude	95% CI	Adjusted	95% CI
Overall infection with SARS-CoV-2 variant								
Omicron BA.1	345	2.1	15,723	97.9	1 (Ref.)		1 (Ref.)	
Omicron BA.2	78	3.0	2,535	97.0	1.39	1.09–1.77	1.20	0.93–1.54
By vaccination status^c^								
**None or only one dose**								
Omicron BA.1	104	3.1	3,228	96.9	1 (Ref.)		1 (Ref.)	
Omicron BA.2	27	4.5	579	95.5	1.43	0.94–2.16	1.37	0.89–2.09
**Two doses**								
Omicron BA.1	155	1.5	10,402	98.5	1 (Ref.)		1 (Ref.)	
Omicron BA.2	28	1.8	1,497	98.2	1.25	0.84–1.86	1.23	0.82–1.85
**Three doses**								
Omicron BA.1	86	3.9	2,093	96.1	1 (Ref.)		1 (Ref.)	
Omicron BA.2	23	4.8	459	95.2	1.21	0.77–1.90	1.00	0.65–1.55

COVID-19: coronavirus disease; IQR: interquartile range; NA: not applicable; Ref.: reference; RR: risk ratio; SARS-CoV-2: severe acute respiratory syndrome coronavirus 2; US: United States.

^a^ Of the 18,760 total cases, 79 were excluded in order to adjust for region, since these cases had missing information for name of region. None of the excluded cases were hospitalised with COVID-19.

^b^ Risk ratios were based on a log-linear Poisson regression model with robust standard errors and calculated using PROC GENMOD in SAS software version 9.4 (SAS Institute, Cary, US). P values for crude and adjusted RR for overall infection with SARS-CoV-2 variant were 0.017 and 0.19, respectively. P values for the stratified analysis represents tests for the interaction between vaccination status and variant (crude RR, p = 0.85; adjusted RR, p = 0.59).

^c^ Among those vaccinated, 86% received the RNA vaccines Comirnaty (BNT162b2 mRNA, BioNTech-Pfizer, Mainz, Germany/New York, United States (US)) or 12% Spikevax (mRNA-1273, Moderna, Cambridge, Massachusetts, US), and less than 2% received the non-replicating viral vector vaccines Vaxzevria (ChAdOx1 nCoV-19, Oxford-AstraZeneca, Cambridge, United Kingdom) or Janssen vaccine (Ad26.COV2-S, Janssen-Cilag International NV, Beerse, Belgium).

A COVID-19 hospitalisation was defined as admission 14 days after or 48 hours before the primary RT-PCR SARS-CoV-2 positive test. All cases were followed for more than 14 days for hospitalisation by using complete admission data extracted on 22 February 2022. Adjusted RRs were adjusted for the basic (a priori) covariates sex, age (10-year groups) [10]. vaccination status (if not stratified by), period as a continuous variable (week 50, 51, 52), region (five groups), comorbidities in the preceding 5 years (none or one or more), and previous SARS-CoV-2 infection within the past 60 days. In a sub-analysis, not shown in the table, we limited COVID-19 hospitalisations to cases with registered COVID-19 diagnoses (diagnostic codes DB342A, DB972A, DB972B, DB948A in the International Classification of Diseases 10^th^ revision) and still did not observe a difference in hospitalisation risk between BA.1 and BA.2 cases (n = 277 hospitalised cases, RR: 1.06 (95% CI: 0.77–1.47).

## Phylogenetic analysis of BA.2

**Figure 1:**
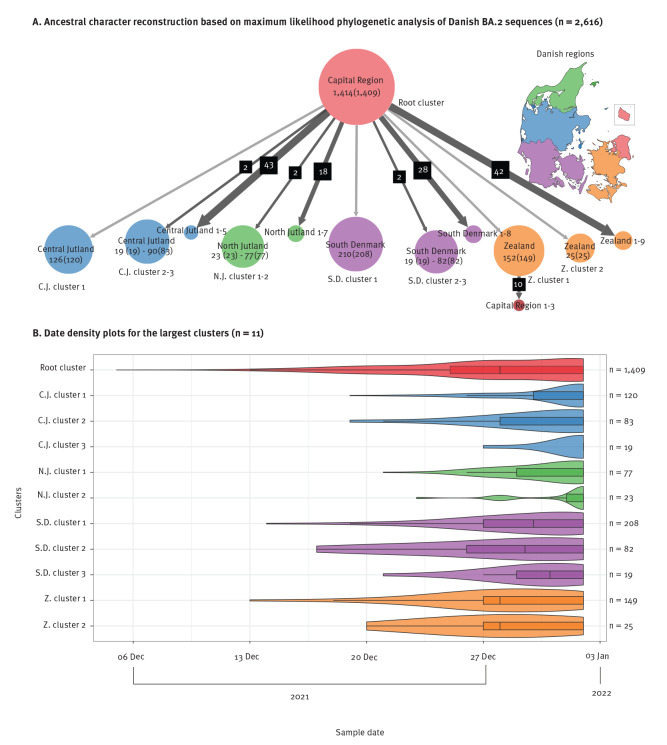
Transmission of SARS-CoV-2 Omicron variant sub-lineage BA.2 across regions and over time, Denmark, 29 November 2021–2 January 2022 (n = 2,616^a^)

BA.2 genomes were aligned using MAFFT version 7.310 with Wuhan-Hu-1 (GenBank accession number: NC_045512.2) as a reference and maximum likelihood (ML) phylogenetic inference was performed using IQ-TREE2, with the transition model, empirical base frequencies and a free rate model with four categories [13,14]. The ML tree (Supplementary data S2: Phylogenetic tree) was rooted with Wuhan-Hu-1 as an outgroup, timescaled and outlier tips removed (seven tips) using Rlsd2 (version 1.10) [15]. Ancestral character reconstruction was performed using PastML (version 1.9.34), with MPPA and F81, annotated to Danish regions [16]. The first introduction of BA.2 was in the Capital Region of Denmark which includes Copenhagen, from where multiple introductions were made to the other four Danish regions in which ten clusters had ten or more samples (Figure 1A). We further delineated clusters with more than 10 samples and visualised them as density plots by sample date using R and ggplot2 (Figure 1B) [17,18]. Three of the clusters (C.J. clusters 1 and 3 and N.J. cluster 2) were characterised by a rapid expansion on 2 January 2022 and one (C.J. cluster 1) also had an expansion on 27 December 2021 (Figure 1B).

C.J.: Central Jutland; N.J.: North Jutland; S.D.: South Denmark; SARS-CoV-2: severe acute respiratory syndrome coronavirus 2; Z.: Zealand.

^a^ Seven sequences were removed as outliers.

A. Ancestral character reconstruction based on maximum likelihood phylogenetic analysis of 2,616 Danish BA.2 sequences. Circles are coloured according to region (see map inset) and the circle size is based on the total number of sequences within each cluster. The numbers in parentheses represent sequences in each cluster after subtrees and missing data were removed. Numbers on the arrows (black boxes) indicate multiple independent transmission events leading to clusters of similar size, with the number of sequences denoted by the numeric range below or next to the region the cluster represents. Note that smaller clusters of multiple transmission events may denote many sequences in the tree, but the circles are scaled according to the size of each individual transmission event, e.g. the 10 transmission events from Zealand to the Capital Region cluster of size 1-3 contains 16 sequences total. Delineation shows the earliest BA.2 sequences of the root cluster in the Capital Region with frequent transmission to all regions. The delineation of transmission only contains Danish BA.2 sequences (Supplementary data S2: Phylogenetic tree) as few BA.2 sequences outside Denmark were available at the time of the study. The paucity of non-Danish BA.2 sequences precluded a precise analysis of the effect of multiple introductions of BA.2 into Denmark. Eleven clusters contained more than 10 sequences.

B. Date density plots for the 11 largest clusters. The plots show sample dates for 11 clusters with more than 10 sequences, with sequences in subtrees and missing geographical data removed. Boxplots show the median sample date for the clusters. For clusters C.J. 1–3 and N.J.1–2, the median day is on 2 December 2022.

## Comparative mutation profiles and structures of BA.1 and BA.2

**Figure 2:**
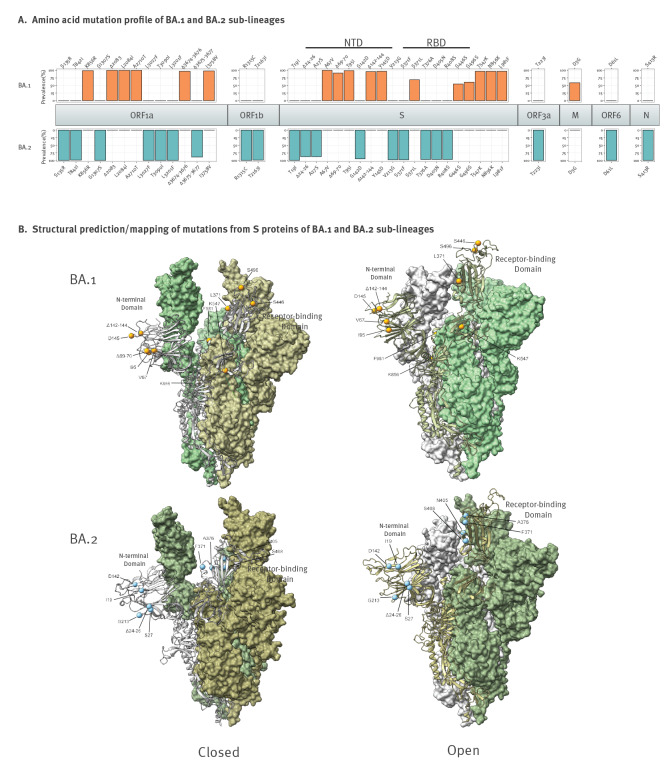
Mutation profile and structural prediction of SARS-CoV-2 Omicron variant sub-lineage BA.1 (n = 16,137) and BA.2 (n = 2,623) sequences, Denmark, 29 November 2021–2 January 2022

NTD: N-terminal domain; RBD: receptor-binding domain; ORF: open reading frame; SARS-CoV-2: severe acute respiratory syndrome coronavirus 2.

Lineage-specific mutations were derived from analysis of WGS consensus genomes with Nextclade CLI 1.9.0 [19] relative to the Wuhan-Hu-1 reference genome and filtered for substitutions and deletions which comprise at least 50% of one lineage and less than 5% of the other. Thirty-nine substitutions and deletions differed between BA.1 and BA.2 and were distributed across the genome (Figure 2A). BA.2-specific spike mutations were clustered in the N-terminal domain (NTD) and in the receptor-binding domain (RBD). BA.1 and BA.2 diverged at spike residue 371 (L and F, respectively) and 142–145 in the RBD. The prevalence of these mutations was close to 100%, except for a few sites with amplicon dropout. A full table of mutations is in Supplementary data S3: Mutation prevalence.

A. Amino acid mutation profile of BA.1 and BA.2 sub-lineages. Mutually exclusive amino acid substitutions and deletions were observed in at least 50% of genomes in this study from either Omicron BA.1 (top orange bars) or BA.2 (bottom blue bars) and not observed more than 5% in the other lineage, from all consensus genomes in study period. Mutations were derived from consensus genomes by use of Nextclade CLI (https://clades.nextstrain.org) [19]. NTD and RBD of the S protein are denoted by bold black lines. Open reading frames in which no exclusive mutations were detected are not shown. Y axes denote prevalence (%) among all genomes for a given lineage and take amplicon dropout into account.

B. Structural prediction/mapping of mutations from S proteins of BA.1 and BA.2 sub-lineages. Structural homology models representing S proteins were generated in SWISS-MODEL using existing template structures (PDB ID: 7KRS, 7T9K) and consensus amino acid spike sequences of BA.1 (top) and BA.2 (bottom) genomes in this study. Representation of trimers shown in closed and open conformations. Substitutions and deletions exclusive to each lineage are shown for BA.1 (orange spheres) and BA.2 (blue spheres) on ribbon representations in each trimer. All visualisations and annotations performed in UCSF ChimeraX (http://www.rbvi.ucsf.edu/chimerax).

Homology models containing both closed and open spike protein conformations were generated in SWISS-MODEL [20] using existing and consensus template structures (https://www.rcsb.org; IDs: 7KRS and 7T9K for BA.1 and BA.2, respectively) and annotated in UCSF ChimeraX 1.2 [21-23] with substitutions and deletions specific to BA.1 and BA.2 (Figure 2B). BA.2 substitutions S371F, T376A, D405N and R408S were seen in the interior RBD surface in both closed and open spike conformations, within the cavity formed by the trimer and also close to an adjacent monomer.

## Ethical statement

This study was conducted using data from the Danish COVID-19 surveillance. According to Danish law, ethical approval is not needed for this type of research.

## Discussion

The SARS-CoV-2 variant Omicron BA.2 sub-lineage has spread rapidly since its first detection in Denmark, while the BA.1 sub-lineage, which appeared 2 weeks earlier, has decreased in numbers. However, we do not find BA.2 cases to be significantly different from BA.1 cases with respect to age, sex, SARS-CoV-2 reinfection, hospitalisation or mortality. These findings are consistent with reports from Norway [24] suggesting that BA.2 leads to an equally mild course of disease with COVID-19 as BA.1 compared with the Delta variant.

Our phylogenetic analyses showed that BA.2 spread from the Capital Region in eastern Denmark to the western parts of the country, mainly through 10 transmission clusters with between 19 and 208 people. No large outbreaks were identified among the clusters from available data sources. Three clusters expanded in a manner suggesting association with travel patterns during the Christmas holiday and have led to the seeding of BA.2 in the population within different geographical regions. This pattern of expansion is in contrast to the initial descriptions of BA.1 in Denmark and Norway, where large single outbreaks were seeding events for the transmission and spread of BA.1 [9,25].

The ability of Omicron sub-lineage BA.1 to replace the previously dominant Delta variant has been attributed to immune escape rather than a higher intrinsic transmissibility [26,27], but BA.2 has been shown to be even more transmissible than BA.1 [10]. Our analysis of the mutation profiles showed different constellations of mutations in BA.1 compared with BA.2, and the structural mapping suggests different effects on receptor binding or changes in interaction with adjacent spike monomers. At the NTD, the BA.2-specific substitution T19I abrogates a glycosylation site at N17 [28,29]. Furthermore, deletions from amino acid positions 24–26 (BA.2), 69–70 (BA.1), 142–144 (BA.1) as well as an A27S substitution (BA.2) are situated in or close to a known NTD antigenic site [30] and are associated with resistance to neutralising monoclonal antibodies [30].

A limitation of this study is that only WGS could be used to identify BA.2, but not all samples were sequenced during the study period because of the high incidence of Omicron in the population at the time. In addition, variant-specific PCR was implemented at different local hospitals but used differentially over time in the community testing centres, which might have affected the pre-selection of samples sent for WGS to some degree. Furthermore, some of the hospital cases might have been admitted for other reasons than COVID-19 and incidentally been detected as part of routine screening of hospital admissions.

## Conclusion

SARS-CoV-2 variant Omicron BA.2 has quickly become the dominant sub-lineage in Denmark, but based on data available on 10 January 2022, BA.2 is not associated with increased severity of disease or hospitalisation. The initial spread of BA.2 in Denmark was characterised by an initial increase in the Capital Region followed by transmission and expansion to the rest of Denmark. The mutation profiles of BA.1 and BA.2 differ in the spike gene in regions associated with receptor binding, glycosylation and resistance to monoclonal antibodies. This study provides novel information about molecular and epidemiological aspects of BA.2 severity, possible national transmission patterns and mutational profile, which can help to inform public health decisions regarding the handling of this Omicron sub-lineage.

## Supplementary Data

685000 Supplement
