# Supplementary material for: Molecular epidemiology of the SARS-CoV-2 variant Omicron BA.2 sub-lineage in Denmark, 29 November 2021 to 2 January 2022
Source: Euro Surveill. 2022 Mar 10;27(10):2200181. doi: 10.2807/1560-7917.ES.2022.27.10.2200181 (PMC8915403; doi:10.2807/1560-7917.ES.2022.27.10.2200181)
Supplement: Supplement [file 22-00181_FONAGER_Supplement.pdf]

This supplementary material is hosted by *Eurosurveillance* as supporting information alongside the article "Molecular epidemiology of the first 2,623 cases of the SARS-CoV-2 variant Omicron BA.2 sub-lineage in Denmark, 29 November 2021 to 2 January 2022" on behalf of the authors who remain responsible for the accuracy and appropriateness of the content. The same standards for ethics, copyright, attributions and permissions as for the article apply. Supplements are not edited by Eurosurveillance and the journal is not responsible for the maintenance of any links or email addresses provided therein.

S1. Lineage Prevalence. Prevalence of the top 10 lineages observed from 120,510 sequenced samples Denmark in the extended study period, 29 November 2021 (week 48) to 6 February 2022 (week 5).

Lineage	WHO classification	2021-W48	2021-W49	2021-W50	2021-W51	2021-W52	2022-W01	2022-W02	2022-W03	2022-W04	2022-W05
Number of sequenced samples [a]	NA	18,357	14,032	7,681	5,388	11,030	9,625	13,754	16,054	13,244	10,985
Sequenced of all RT-PCR positive samples (%) [b]	NA	52.8	28.8	10.1	6.7	8.7	7.0	7.5	5.6	4.4	3.7
BA.1 (%)	Omicron	2.7	15.2	55.4	71.9	68.9	60.0	39.0	23.6	13.3	7.0
BA.2 (%)	Omicron	<0.1	<0.1	2.7	9.6	20.5	31.1	53.8	70.3	81.9	89.2
BA.1.1 (%)	Omicron	<0.1	0.2	0.7	1.7	5.1	6.4	6.5	5.9	4.8	3.8
AY.43 (%)	Delta	24.6	21.8	11.4	4.4	1.5	0.8	0.2	<0.1	<0.1	0
AY.4 (%)	Delta	26.1	23.0	10.3	4.5	1.5	0.6	0.2	0.1	0	<0.1
AY.122 (%)	Delta	15.8	13.6	6.3	2.3	0.8	0.3	0.1	<0.1	<0.1	0
AY.4.2 (%)	Delta	6.6	7.0	3.7	1.6	0.5	0.3	0.1	<0.1	<0.1	0
AY.127 (%)	Delta	4.4	3.4	1.7	0.6	0.3	0.2	<0.1	<0.1	0	0
AY.43.6 (%)	Delta	9.2	7.4	3.9	1.5	0.4	0.2	0.1	<0.1	<0.1	0
AY.126 (%)	Delta	10.6	8.4	3.9	1.7	0.4	0.2	<0.1	<0.1	0	<0.1

Legend: a: total number of sequenced samples from cases with genome data (low quality genomes excluded) within the week. b: percentage of sequenced samples with genome data out of the total number of SARS-CoV-2 RT-PCR positive samples within the week. All other entries show the percentage of sequenced samples belonging to a lineage among all samples with genome data (low quality genomes excluded) per week. Omicron designations (B.1.1.529, BA.1 and BA.2) are performed by Pangolin based on the mutational profiles of each lineage/sublineage. Data for the extended study period, used only in this table, was extracted on 16 February 2022.

S2. Phylogenetic tree. Maximum likelihood phylogenetic tree annotated to Danish regions

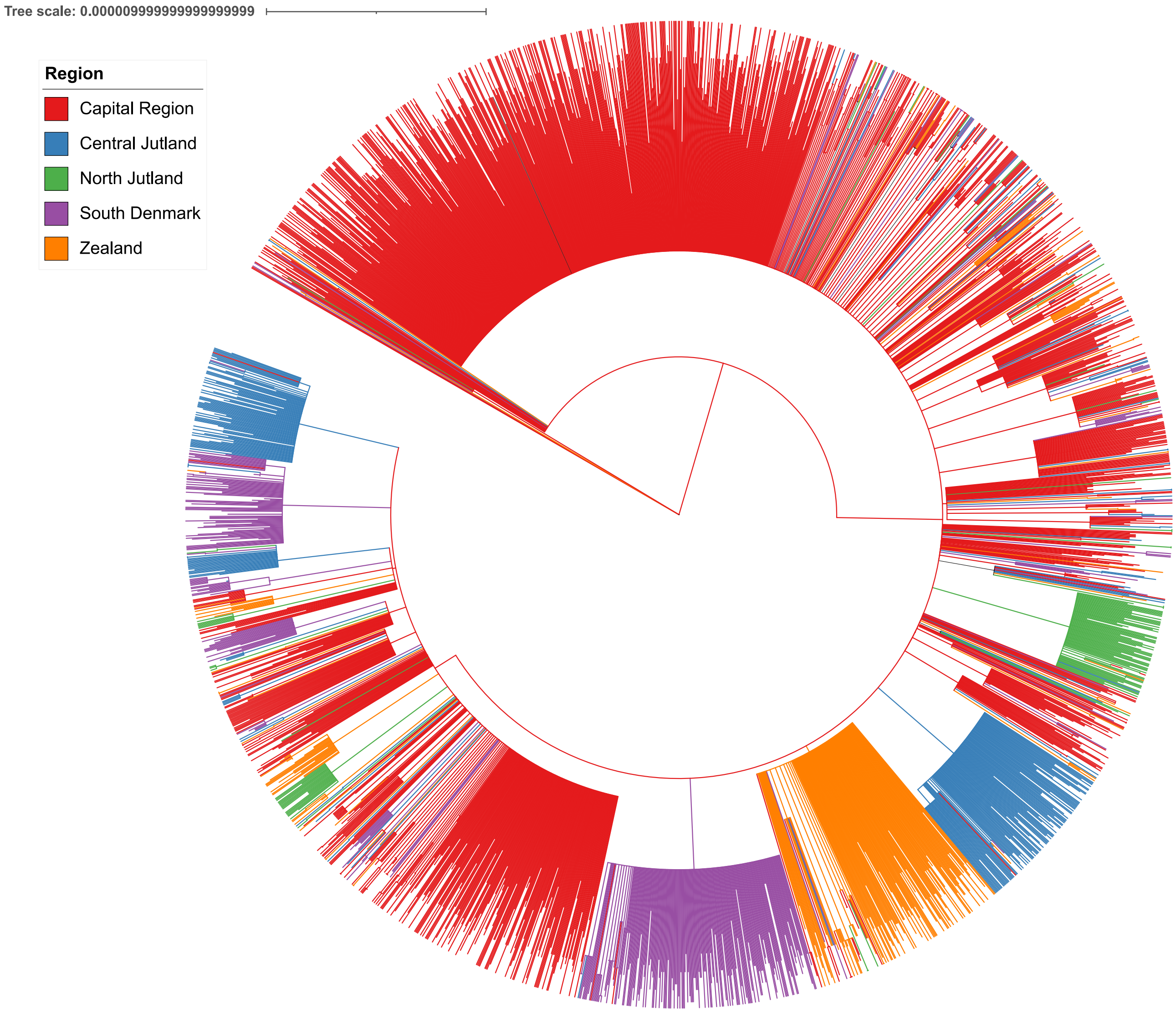

S3. Mutation prevalence. All mutations observed in 18,760 BA.1 and BA.2 sequenced samples during the study period 29 November 2021 (week 48) to 2 January 2022 (week 52).

AA Mutations	BA.1 Prevalence(%)	BA.2 Prevalence(%)
ORF1a:S135R	0,0	99,8
ORF1a:T842I	0,0	99,5
ORF1a:K856R	99,5	0,0
ORF1a:G1307S	0,0	99,8
ORF1a:Δ2083	100,0	0,0
ORF1a:L2084I	99,6	0,0
ORF1a:A2710T	99,7	0,0
ORF1a:L3027F	0,0	99,5
ORF1a:T3090I	0,0	99,5
ORF1a:L3201F	0,0	99,6
ORF1a:T3255I	99,9	99,7
ORF1a:P3395H	99,7	99,9
ORF1a:Δ3674-3676	97,0	0,0
ORF1a:Δ3675-3677	0,0	89,0
ORF1a:I3758V	98,1	0,0
ORF1b:P314L	99,9	99,9
ORF1b:R1315C	0,0	99,9
ORF1b:I1566V	99,9	99,9
ORF1b:T2163I	0,0	99,4
E:T9I	99,9	99,9
S:T19I	0,0	99,0
S:Δ24-26	0,0	87,0
S:A27S	0,0	87,5
S:A67V	99,6	<5.0
S:Δ69-70	91,0	0,0
S:T95I	98,8	0,0
S:G142D	0,0	94,2
S:Δ142-144	96,0	0,0
S:Y145D	96,9	0,0
S:N211-	45,0	0,0
S:L212I	42,6	0,0
S:V213G	0,0	97,6
S:G339D	95,0	96,9
S:S371F	<5.0	96,8
S:S371L	70,3	<5.0
S:S373P	72,4	96,9
S:S375F	72,7	96,8
S:T376A	<5.0	96,4
S:D405N	<5.0	96,7

S:R408S	<5.0	96,4
S:K417N	65,2	96,3
S:N440K	51,4	88,6
S:G446S	57,7	0,0
S:S477N	54,9	94,0
S:T478K	55,8	94,1
S:E484A	55,6	94,9
S:Q493R	60,6	95,8
S:G496S	62,7	0,0
S:Q498R	63,1	96,7
S:N501Y	63,7	96,4
S:Y505H	61,3	96,4
S:T547K	96,8	0,0
S:D614G	97,3	99,8
S:H655Y	97,3	99,9
S:N679K	97,2	99,9
S:P681H	97,2	99,8
S:N764K	95,3	98,7
S:D796Y	96,2	99,6
S:N856K	97,2	0,0
S:Q954H	97,2	99,8
S:N969K	97,1	99,9
S:L981F	97,1	0,0
M:D3G	60,5	0,0
M:Q19E	88,5	78,5
M:A63T	99,9	99,8
ORF3a:T223I	0,0	99,8
ORF6:D61L	<5.0	99,5
N:P13L	97,3	98,5
N:Δ31-33	96,0	97,0
N:R203K	98,4	98,7
N:G204R	98,4	98,9
N:S413R	0,0	99,2
ORF9b:P10S	97,2	98,5
ORF9b:Δ27-29	96,0	97,0

Legend: All amino acid mutations (SNPs and deletions) observed in BA.1 or BA.2 consensus genomes generated during the study period. Mutations were derived using Nextclade CLI (*clades.nextstrain.org*) and processed with a custom Perl script.
